# The Local Government Board and Guardians' Contracts

**Published:** 1915-08-21

**Authors:** 


### POOR-LAW MEDICAL APPOINTMENTS.

### The Local Government Board and Guardians' Contracts.

The appointment of a Departmental Committee by the Local Government Board to inquire into the provisions of the Poor-Law Orders on the appointments, qualifications, tenure of office, duties, and remuneration of certain Poor-Law officers, and the recent dismissal by the Epping Guardians of their medical officer—a matter upon which we have commented several times in our Notes—render the conditions of appointment of Poor-Law medical officers of special interest at the present time. In the first Annual Report of the Poor-Law Commissioners, issued in 1835, the Guardians are recommended to ask the medical practitioners in the Union to compete for the post of medical officer and to appoint the one who tendered for the work at the lowest price. This principle when acted upon was found to give rise to so many scandalous results that the Commissioners were soon compelled to modify their views of the value of cheap medical attendance. Consequently, when they drew up the Consolidated Orders in 1847 a clause was inserted forbidding the Guardians to invite tenders for medical attendance on the paupers of the Union, and requiring them to pay such salaries or remuneration as the Commissioners directed or approved. At the same time it was ordered that every medical officer should, unless the period of his appointment was entered on the minutes of the Guardians at the time, or acknowledged in writing by him, continue in office until he died, resigned, became legally disqualified to hold office, or was removed therefrom by the Commissioners. It was found that this clause was not stringent enough to ensure the security of tenure of office necessary if the medical officer was to perform his duties faithfully.

The clause was therefore rescinded, and in 1857 the following Order was issued:—" Every Medical officer duly appointed shall hold his office until he shall die, or resign, or be proved insane by evidence which the Poor-Law Board shall deem sufficient, or become legally disqualified to hold such office, or be removed by the Poor-Law Board." This is the Order which still governs all appointments of district or workhouse medical officers under the Poor Law.

Many Boards of Guardians have tried in various ways to evade this regulation. In many Unions, under various pretexts, attempts have been made to make the appointment of district medical officers an annual one. The Local Government Board has always refused its sanction to such an arrangement unless good reasons, such as the possible reorganisation of the medical service of the Union in the near future, could be adduced in support. Another method which has been adopted in many instances has been to include in the contract signed by the medical officer a clause making the appointment terminal by notice on either side. To the surprise of Poor-Law officials, the Local Government Board has never taken any exception to these contracts. As we pointed out last week, the Board has now ruled that such a clause can affect only the term of the contract itself and cannot apply to the appointment. Undoubtedly, however, both Boards of Guardians and medical officers have considered these contracts gave the Guardians the power to terminate the appointment upon the agreed notice The result has been to deprive the medical officer of the feeling of security in his office which the Order of 1857 was meant to give him, so long as his work was conscientiously and faithfully performed. Now, however, that the Local Government Board has made it clear that such contracts have no effect as against the Board's regulations, we shall probably hear no more of them.

It is unfortunate that in the case of the separate infirmaries the Local Government Board has in recent years slightly modified the Order of 1857, to the detriment of the medical officer. Under that Order a medical officer can only be dismissed by the Local Government Board. Under the Orders issued to individual separate infirmaries during the last few years the medical superintendent may be dismissed by the Guardians subject to the consent of the Local Government Board. In practice this change might be much more detrimental to the interests of the medical officer than would appear at first sight. Under the Order of 1857 the Guardians, if they wish to dismiss their medical officer, must first place their reasons before the Local Government Board. Under these infirmary regulations the Guardians would start with the advantage of an accomplished fact. It is the difference between standing on trial for an alleged offence and appealing against a conviction.

### Security of Tenure.

In the evidence that Poor-Law medical officers will no doubt place before the Departmental Committee through their Association, it is to be hoped that stress will be laid upon the importance of maintaining security in their tenure of office. It is not a question of attempting to condone offences when offences have been committed. Under such circumstances everyone would desire that the Local Government Board should exercise its powers and dismiss the medical officer. It is a question of protecting the medical officer who, in the conscientious performance of his duties, finds himself at cross purposes with his Board. An equally unfortunate matter is that the Local Government Board should lay down minimum salaries based upon the amount of work required, with provision for periodic increases. In default of such a scale Guardians in the past have found it easy to take advantage of the competition amongst medical men, the results of which were found so detrimental when appointments were given to the lowest bidder. And in the absence of provision for periodic increases Guardians have found a means of punishing medical officers who have earned them, but have incurred the displeasure of their Board in the conscientious performance of their work. Another important alteration in the terms of appointment, and one towards which the Local Government Board is favourably inclined, is the prohibition of any contract requiring the medical officer to supply the drugs needed for the treatment of his patients. This system was unreservedly condemned by the last Royal Commission on the Poor Laws, and the Local Government Board and its inspectors have frequently drawn the attention of Boards of Guardians to the defects inherent in it.

